# Positive-negative tunable liquid crystal lenses based on a microstructured transmission line

**DOI:** 10.1038/s41598-020-67141-z

**Published:** 2020-06-23

**Authors:** J. F. Algorri, P. Morawiak, N. Bennis, D. C. Zografopoulos, V. Urruchi, L. Rodríguez-Cobo, L. R. Jaroszewicz, J. M. Sánchez-Pena, J. M. López-Higuera

**Affiliations:** 1 Photonics Engineering Group, University of Cantabria, 39005 Santander, Spain; 2 New Technologies and Chemistry Faculty, Military University of Technology, Warsaw, 00-908 Poland; 3 Consiglio Nazionale delle Ricerche, Istituto per la Microelettronica e Microsistemi (CNR-IMM), Rome, 00133 Italy; 4 Department of Electronic Technology, Carlos III University, Madrid, 28911 Spain; 5 CIBER-bbn, Instituto de Salud Carlos III, 28029 Madrid, Spain; 6 Instituto de Investigación Sanitaria Valdecilla (IDIVAL), 39011 Santander, Spain

**Keywords:** Applied optics, Adaptive optics

## Abstract

In this work, a novel technique to create positive-negative tunable liquid crystal lenses is proposed and experimentally demonstrated. This structure is based on two main elements, a transmission line acting as a voltage divider and concentric electrodes that distribute the voltage homogeneously across the active area. This proposal avoids all disadvantages of previous techniques, involving much simpler fabrication process (a single lithographic step) and voltage control (one or two sources). In addition, low voltage signals are required. Lenses with switchable positive and negative focal lengths and a simple, low voltage control are demonstrated. Moreover, by using this technique other optical devices could be engineered, e.g. axicons, Powell lenses, cylindrical lenses, Fresnel lenses, beam steerers, optical vortex generators, etc. For this reason, the proposed technique could open new venues of research in optical phase modulation based on liquid crystal materials.

## Introduction

The use of liquid crystal (LC) materials for non-display applications has been extensively researched. Specifically, LC-tunable optical phase modulation is one of the most important uses, which has attracted significant attention for years^1^. Currently, it is a very researched topic, for example in ophthalmological applications^2^, 3D vision applications^3–6^, beam steering^7^, optical vortices^8–11^, tunable zooming^12^, aberration correctors^13^, astronomy^14^, novel aberration correctors^15^, multi-optical elements^16^, micro-axicon arrays^17^, multi-focal^18^, high fill-factor^19^ and frequency controlled^20^ microlenses, lensacons, and logarithmic axicons^21^, etc. High among those are adaptive-focus lenses, which were first proposed more than 40 years ago and still are a hot topic. Some of the first structures were proposed by Berreman *et al*. (with patent application in 1977^22^) and Sato *et al*. in 1979^23^ at the end of the seventies. In that case, the topology was based on curved cavities filled with LC. There were some important problems such as low response time, due to the increased LC layer thickness, and the molecular orientation inhomogeneity. Then, the first cylindrical LC lens was demonstrated in 1981^24^. Several electrodes were used in order to obtain the proper voltage gradient. This new concept of lens stimulated other research works^25^ and subsequently spherical lenses^26^. At the end of the eighties, LC lenses at micrometric scale were demonstrated^27,28^. In addition, the first Fresnel lenses were reported on those years^29^. The main advantage is the possible high optical power, reducing the necessary thickness and enabling large apertures. Also in the eighties, an ingenious design that allows large apertures was proposed by A.F. Naumov *et al*.^30^. Namely, the use of high resistivity layers in order to avoid the voltage drop at the edges^31^. One of the most important parameters is the sheet resistance of the control electrode. As the diameter increases, the required sheet resistance decreases. Some typical values can be 100 kΩ/sq to few MΩ/sq for lens diameters of several millimeters^32^. Thanks to the use of this technique, other devices and applications has been proposed, e.g. wavefront correctors^13,33^, microlenses^34^, multioptical devices^35,36^ or optical tweezers^37,38^. Other techniques to fabricate LC lenses have been proposed in recent years. For example, photoalignment and photopatterning are also important techniques^39–41^. In order to have a general vision of all of them, we recommend the review on LC lenses by Yi-Hsin Lin *et al*.^42^. For more specific topics, the reader can find some useful reviews, e.g. fast-response time LC microlenses^43^, LC microlenses for autostereoscopic displays^44^, design and fabrication^45^, LC contact lenses for the correction of presbyopia^46^ or recent advances in LC lenses^47^. Although the main structures were established several years ago, there is still room for new proposals. Specifically, for the case of large apertures the modal and multielectrode topologies have been the most successful. The modal technique has been able to produce lenses of several millimeters with good homogeneity (up to 5–10 mm) and low voltage control (few volts). The main disadvantage is the complex fabrication process due to the difficulty to obtain homogenous high resistivity layers as the diameter increases. On the other hand, multi-electrode lenses do not face this problem, as the electrodes are etched in commercial indium titanium oxide (ITO). Nevertheless, the complexity comes with the voltage control, as the number of electrodes increases the required contacts and a proper voltage controller can be extremely difficult to implement.

In this work, we propose a novel ITO-on-glass micrometric structure capable of creating large aperture lenses with simple voltage control and homogenous distribution of the voltage. The device operates similarly to multielectrode and modal lenses, but without the disadvantages commented before. Moreover, the proposed transmission line LC lenses can have both positive and negative focal lengths, thus doubling the possible optical power.

## Structure and operating principle

**Figure 1:**
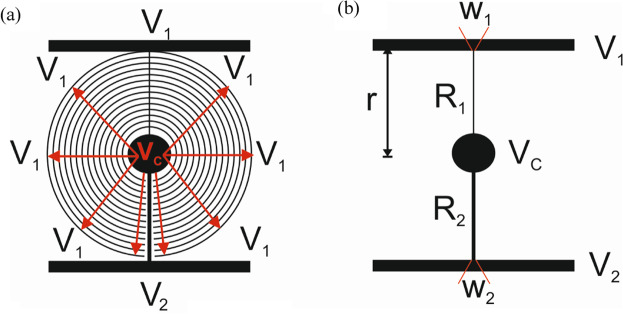
Schematic depiction of the (**a**) entire electrode configuration structure (*V*_*C*_ in red denotes central voltage) and (**b**) transmission line without the concentric electrodes.

Considering the previous statements, it is required to find a specific design capable of producing a high optical power with a simple design and voltage control. In order to obtain such device, only one lithographic step and high reproducibility are desired. For these reasons, the best option is to use an ITO layer behaving like a high resistivity layer. Some works have proposed the use of thin ITO layers, despite this, obtaining such thin layers is very complicated. The idea behind this work, is based on micrometric gaps that make a commercial ITO substrate behave as high resistivity layer, without the disadvantage of complex fabrication. This micrometric structure is based on two main elements, a transmission line, Fig. 1(a), acting as a voltage divider, and concentric electrodes, Fig. 1(b), which distribute the voltage homogeneously across the active area. It is important to note that concentric electrodes make electrical contact with the thinner transmission line (*R*_*1*_) only and do not contact *R*_*2*_.

The main objective is to control the voltage distribution between *V*_*1*_ and *V*_*C*_ in order to work between threshold and saturation voltages of the LC tunable birefringence curve. In the next sections, each component is explained in detail.

**Figure 2:**
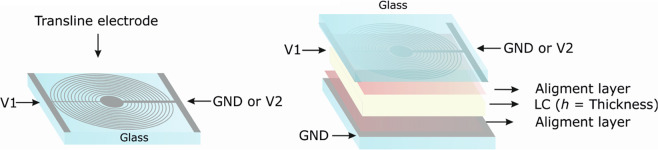
Diagram of the proposed device.

The micrometric structure is patterned on a commercial ITO over glass substrate. This substrate is placed in the upper part on Fig. 2. As can be observed, the bottom substrate does not require any patterning; it is a continuous ITO electrode. The device is simple, i.e. two substrates with electrodes, which form a planar LC cell with standard alignment layers to control the LC molecular orientation at the interfaces.

### Transmission line

Taking into account the threshold and saturation voltage of the LC, the transmission line is designed such that it can distribute the voltage accordingly. In order to obtain both positive and negative focal lengths, two voltage sources are required. The transmission line acts as a classical voltage divider. Due to the low resistivity of the transmission line, the voltage distribution is simply governed by Ohm’s law (*V* = *I* ∙ *R*). By considering the structural characteristics of the transmission line (length, width and *R*_*sq*_) the total resistance for each branch (*R*_*1*_ and *R*_*2*_) can be obtained (*R* = *R*_*sq*_ ∙ length/width). Then, the resulting voltage at the center (*V*_*C*_) can be estimated by using Eq. 1.

1 $${V}_{C}=({V}_{1}-{V}_{2})\frac{{R}_{2}}{{R}_{1}+{R}_{2}}+{V}_{2}$$

**Figure 3:**
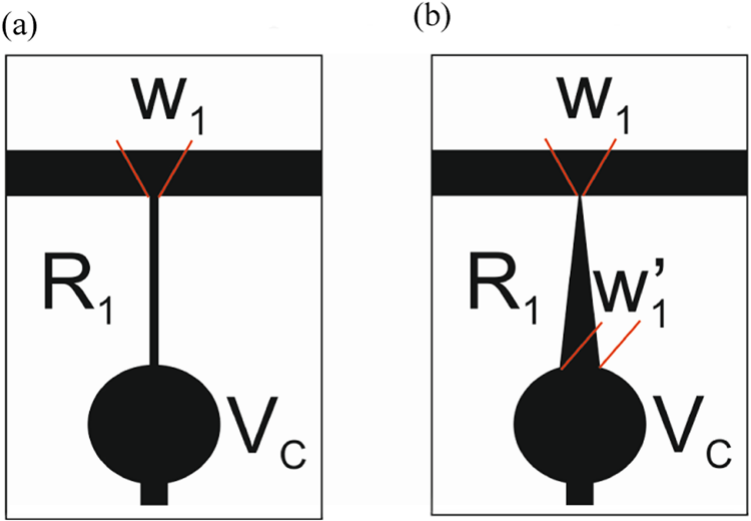
Schematic depiction of the transmission line *R*_*1*_ with (**a**) constant resistance, (**b**) linear resistance.

In contrast with the modal technique, this transmission line has low resistivity but high resistance, due to the thin width in comparison with the length. It has to be considered that this resistance is higher in the space between concentric electrodes. For this reason, the total resistance *R*_*1*_, can be estimated considering the resistance between concentric electrodes multiplied by the number of gaps. As can be seen in Fig. 3, there is a circle at the center that separates *R*_*1*_ and *R*_*2*_. The diameter of this circle will depend on the required lens flatness at the center. It has to be considered then, that the length of *R*_*1*_ and *R*_*2*_, is the lens radius minus the center circle radius. On the other hand, to obtain parabolic voltage profiles across the transmission line, the resistance *R*_*1*_ can be designed to decrease linearly when approaching the center of the lens (in the numerical example *W*_*1*_ = 1 µm and *W*_*1*_′ = 10 µm).

The required voltages for *V*_*1*_ and *V*_*2*_ are considered by using Eq. 1. In this case the parameters for a constant resistance are *W*_*1*_ = 10 µm, *W*_*2*_ = 60 µm and *R*_*sq*_ = 100 Ω/sq (for this example the effect of the concentric electrodes and the circle at the center are not considered). The desired values for *V*_*1*_ and *V*_*C*_ are fixed for a desired LC profile whereas *V*_*2*_ is the unknown variable. Here, we aim at voltage gradients between 1 V and 3 V on *V*_*1*_ and *V*_*C*_, targeting both positive and negative phase profiles. The applied voltages at *V*_*2*_ for eight configurations in total (four positive and four negative) are calculated by Eq. 1 as explained and summarized in Table 1.

**Table 1:** Required voltages for eight different positive and negative phase profiles.

V_1_	V_C_	V_2_	Conf.	Type
1	1.5	1.58	a	Positive
1	2	2.17	b	
1	2.5	2.75	c	
1	3	3.33	d	
1.5	1	0.92	e	Negative
2	1	0.83	f	
2.5	1	0.75	g	
3	1	0.67	h	

**Figure 4:**
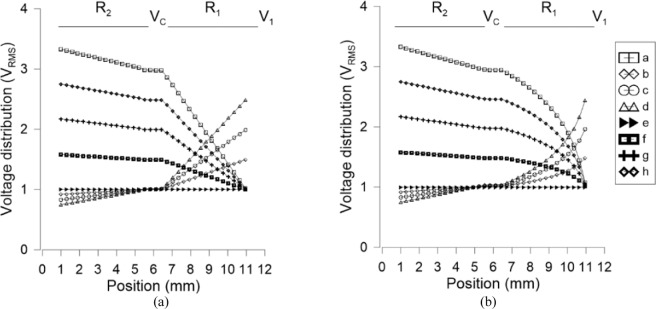
Simulation of the voltage distribution across the transmission line for (**a**) constant resistance, (**b**) linear resistance. Applied voltages are described in Table 1.

The voltage distribution along the electrode has been solved by the finite element method commercial tool COMSOL Multiphysics. By applying the estimated voltages of Table 1 in the proposed devices, the voltage gradient is numerically solved. As can be observed in Fig. 4, the voltage profile can be linear or parabolic depending on the topology used, namely rectangular or triangular shapes shown in Fig. 3(a,b), respectively. Other voltage profiles could be possible by modifying the shape of the electrode *R*_*1*_. An optimization of the electrode shape brings the possibility of perfect lenses (without aberrations), but this hypothesis has to be further investigated.

In order to take advantage of the profile produced in *R*_*1*_ electrode (between *V*_*1*_ and *V*_*C*_), a series of concentric electrodes are connected to this line in order to distribute the voltage across the lens surface.

### Concentric electrodes

**Figure 5:**
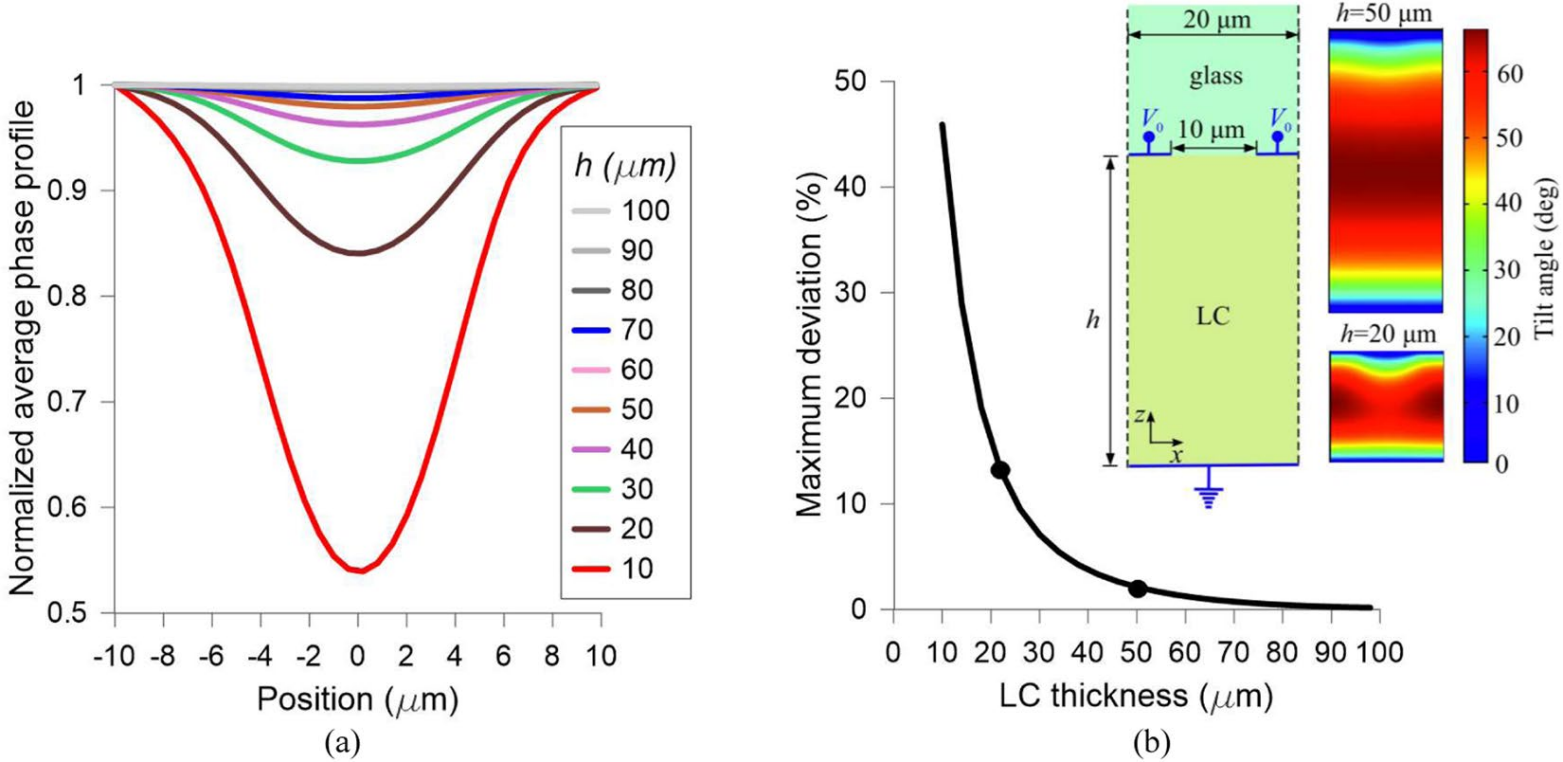
Simulation of the phase shift distribution between concentric electrodes (**a**) for several LC thickness and normalized to the value of the phase shift on the electrode (**b**) maximum deviation from the electrode to the middle of the space between electrodes, and an inset showing the LC molecular distribution for 2 thickness values, 20 µm and 50 µm.

The concentric electrodes (crossing perpendicular to the transmission line), distribute the voltage across the active area (acting equivalently to the high resistivity layer in a modal lens). These electrodes are only connected to *R*_*1*_. The space between electrodes is a critical parameter in order to avoid steep variations of the phase in these regions. For this reason, we estimate the effect of the gap between adjacent electrodes on the uniformity of the LC tuning profile by investigating the structure shown in the inset of Fig. 5(b). The LC is confined in a cell of thickness *h* and homogeneously aligned along the y-axis with a pretilt angle of 1°. The classic E7 LC material is used for the simulations. The bias voltage is applied between the grounded bottom and the top electrode, which is half the total pitch of *p* = 20 μm. A top glass layer supports the LC cell and the structure is assumed periodic laterally. The inter-electrode gap leads to less average tuning in the mid-zone of the cell, which is more profound for LC cells with lower aspect ratios *h/p*. To quantify this effect, we calculate first the local LC refractive index *n(x,z)* sensed by y-polarized light, which is given by^48^

2 $${n}_{av}(x,z)=\sqrt{\frac{{n}_{o}^{2}\cdot {n}_{e}^{2}}{{n}_{o}^{2}{\cos }^{2}\theta (x,z)+{n}_{e}^{2}{\sin }^{2}\theta (x,z)}}.$$

Then, we calculate the average index along the cell as

3 $${n}_{av}(x,z)={\int }_{0}^{h}n(x,z)dy/h$$

For *V*_*0*_ = 0 V the effective index approaches the LC extraordinary index. Therefore, the average index modulation between zero and a given voltage is *Δn*_*av*_*(x)* = *n*_*e*_ - *n*_*av*_*(x)* and the corresponding phase modulation *Δφ(x)* for a y-polarized plane wave propagating along the z-axis is proportional to *Δn*_*av*_*(x)*, which is maximized at the mid-point of the electrode width.

Figure 5(a) plots the normalized *Δφ(x)* for LC thickness values from 10 to 100 μm and *V*_*0*_= 2V. The maximum relative deviation occurs at the mid-point of the inter-electrode gap, its value exponentially increases to significant values as the aspect ratio *h/p* becomes less than unity, as shown in Fig. 5(b). However, for *h* > 50 μm the maximum deviation is less than 2%, which indicates that the effect of the inter-electrode gap in the LC lenses investigated in the next Sections is marginal since they employ LC cells at least 50 μm thick. Finally, the inset of Fig. 5(b) shows the profiles *θ(x,z)* of the tilt angle in the y-z plane for an indicative applied voltage *V*_*0*_ = 2 V and two values of LC thickness, *h* = 20 and 50 μm.

### Whole structure

Finally, the phase profile produced by this device is estimated by considering the voltage distribution of Fig. 4.

**Figure 6:**
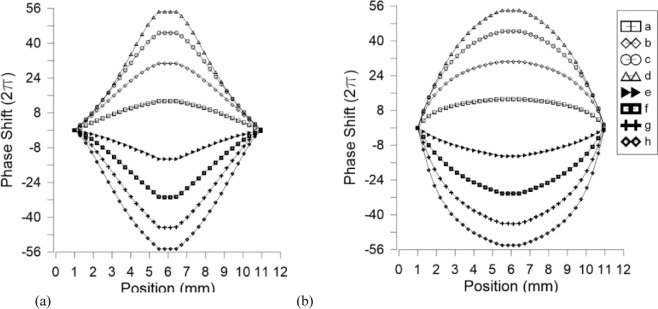
Simulation of the phase shift distribution across the lens surface for (**a**) constant resistance, (**b**) linear resistance. Applied voltages are described in Table 1.

The effective refractive index produced by the molecules is considered in order to estimate the total phase shift produced in the lenses. In order to test the maximum capabilities of this structure, a thickness of 100 µm and a high birefringence LC (LCM-UHB.1865 *Δn@25°* = 0.50^49^) are considered. The refractive indices of this LC material do not appear on ref. ^49^, so typical values of high birefringent LC are used, in this case *n*_*o*_ = 1.5 and *n*_*e*_ = 2. In the case of Fig. 6(a), the voltage is linear at the sides producing axicon-type lenses^21^. When the voltage is high enough a logarithmic lens is produced [see Fig. 6 voltage configuration (d)]^21^. For the case of linear resistance, Fig. 6(b), a quadratic phase profile is obtained. This lens could be aberration-free by controlling the shape of *R*_*1*_. Simulations reveal that positive-negative tunability is possible in both cases by applying two different voltages to electrodes *V*_*1*_ and *V*_*2*_. The maximum phase shift is ±112π, which is equivalent to a focal length of ±35 m or ±2.86D. As a result, the proposed structure could provide a total optical power of 5.7D for an aperture of 1 cm.

## Fabrication

**Figure 7:**
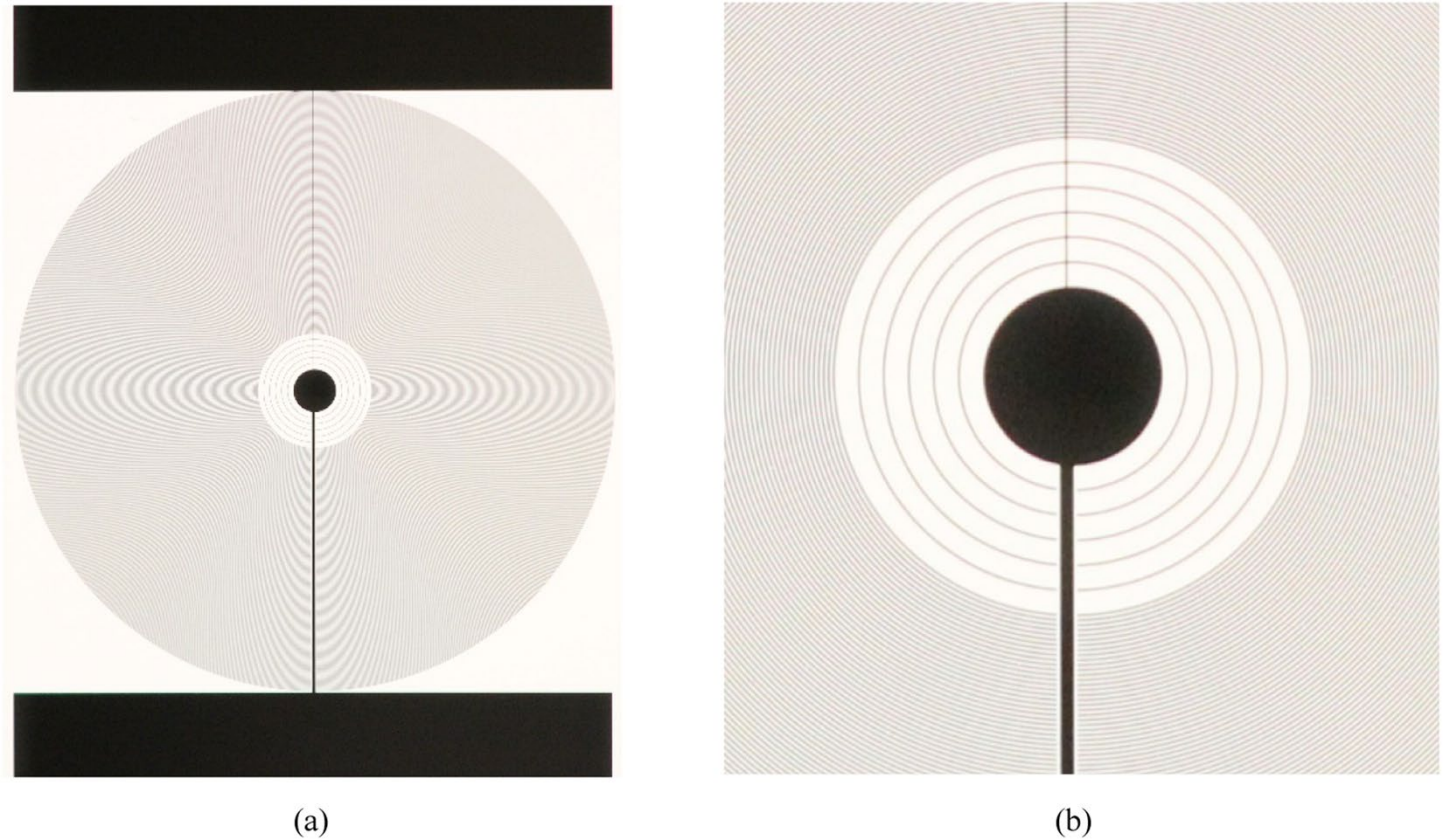
Chrome mask used to fabricate the transmission line of the LC lens, (**a**) whole structure (**b**) detail of the center. In this case the parameters are *W*_*1*_ = 10 µm, *W*_*2*_ = 60 µm, gap = 10 µm. Circle at the center 0.7 mm.

The proposed lens consisted of a liquid crystal layer sandwiched between two glass substrates. This optical device requires transparent electrodes to generate an electric field through the liquid crystal layer. To manufacture this device, a Glaston polished glass (supertwist quality) with thickness 0.7 mm and a 50 nm layer of Indium tin oxide (ITO) is used as the electrode material. The ITO layer is a transparent electrode (80–90%) whose *R*_*sq*_ = 100 Ω/sq. In order to pattern the electrodes on each substrate, a photolithographic process is used to transfer a designed pattern from a chrome photomask (Fig. 7(a)) to a light-sensitive chemical photoresist on the substrate. As can be observed in Fig. 7(b), the circle at the center have a diameter of 1 mm and there are some concentric electrodes with larger gap close to it. The reason of this is that lenses have a considerable low phase shift at the lens center, therefore it is not necessary to have high concentration of electrodes in this region. The concentric electrodes have a width of 10 µm and are separated by a gap of 10 µm.

The photoresist is spun on the substrate at 3500–4000 rpm for 30 seconds. This results in a film thickness of about 2 microns. After spin-coating, the photoresist was then subsequently cured in two stages: 65 °C for 5 min and 90 °C for 30 min. The designed geometry patterns (Fig. 7) were developed using a standard photolithography process upon UV exposition of photoresist through a photomask chrome on glass masks (*λ* = 365 nm, 150 mWcm^−2^ for 5 s). After the exposure, a developing process using Microposit Developer (Shipley Far East Ltd. Japan) leaves the resist only on the desired electrodes. In this process, the illuminated photoresist is removed. This stage is the most difficult step in the photolithographic process since the electrode has a line pattern with different sizes and different gaps between lines. The adhesion of the resist to the surface is critical for high-resolution patterning. However, for the patterns featured here this effect is not noticed. The optimal developing time is obtained after several trial-error tests. This time is inversely proportional to the power of the light illuminating the photoresist and directly proportional to its thickness. The remaining developer is washed away with distilled water. The substrates are dried using a pressurized N2 gun and placed in a hot stage 30 min at 110 °C in order to harden the photoresist. By using an etch bath a wet etching process is carried out, it etches the ITO from the areas not covered by resist. For this process an acid bath of HCl:H_2_O:HNO_3_ heated to 58 °C is used. The optimal etching time is also obtained after several tests due to the complexity of the designed electrodes mentioned above. This time depends on the ITO thickness and the correct proportions of the chemical mixture. Temperature and soaking times are optimized to 58 °C and 65 s respectively. After this time, the substrates have to be rinsed in water fast enough to prevent the acid mixture from undercutting the photoresist layer. Once the ITO has been attacked, the remaining photoresist is removed by using a solvent liquid (Microposit Remover). Substrates are immersed in this bath for 90 seconds and then rinsed. After the patterning process, a polyimide (PI) alignment layer is coated and rubbed on the patterned substrates. After rubbing treatment, the two substrates are attached by using a photo-cured adhesive material deposited at the edges of the cell. The LC cells is assembled by dispensing a photopolymerizable epoxy (NOA 68) perimeter seal on one substrate, while high precision 50 μm silica sphere spacers are dispersed on the opposing substrate. The two substrates are optically aligned facing each other’s interior surface with an opposite rubbing direction. Finally, the nematic LC (6CHBT^50^) is injected into the gap between the two glass via the capillary effect and the liquid crystal is sealed perfectly using the epoxy. The characteristics of 6CHBT at 20 °C: phase transition temperatures: Cr 13 °C N 43 °C Iso, Density: 1.101 g/cm^3^ (at T = 20 °C), Optical refractive indices: n_e_ = 1.68 and n_o_ = 1.51 (*Δn* = 0.17), Dielectric permittivities: ε⊥ = 4.30 and ε_//_ = 12 (at frequencies 1 KHz) and Viscosity = 21mPas at 20 °C.

## Experimental set-up and results

### Experimental setup

**Figure 8:**
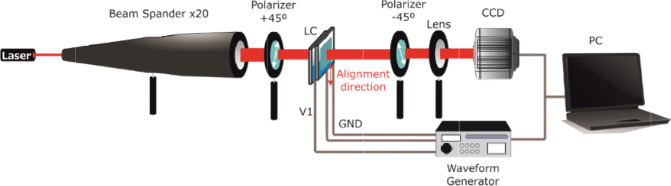
Depiction of the experimental setup.

For the experimental set-up (Fig. 8), a classical interferometric stage is used. A 632.8 nm laser with the beam expanded 20 times is used as a light source. Then, the LC lens is placed between crossed polarizers (the sample at 0°, one polarizer at +45° and other one at −45°).

With this configuration, the ordinary wave is always affected by the ordinary refractive index and is considered as reference. The extraordinary wave is affected differently across the active area due to the molecular switching (*n*_*o*_ for voltages below the threshold and *n*_*e*_ for voltages close to the saturation). The light through regions where the phase shift is even multiple of π is absorbed by the second polarizer, producing minimum transmittance. Phase shifts odd multiple of π pass through and produce maximum transmittance. Thanks to this setup, the phase shift produced by the device is captured in a CCD. Then, the phase profiles are obtained from interference patterns by a specially developed image recognition program based on fringe skeletonizing technique^11^.

### Results

In this section, the operating principle of the transmission line and concentric electrodes is experimentally demonstrated. As commented above, the transmission line produces a linear voltage distribution from one electrode to the other. Then, the concentric electrodes (perpendicular to the transmission line), distribute the voltage across the active area homogeneously. In order to demonstrate the feasibility of the proposed structure, the topology of Fig. 3(a) is fabricated. The parameters are *W*_*1*_ = 10 µm, *W*_*2*_ = 60 µm, gap = 10 µm and *R*_*sq*_ = 100 Ω/sq. The diameter of the circle at the center is 0.7 mm. The measured total resistance between electrode *V*_*1*_ and *V*_*2*_ is 50 kΩ so the expected current is in the order of mA. This resistance is little higher than theoretically estimation, probably caused by an increase of the sheet resistance in the acid attack. The electrode *R*_*1*_ has a constant resistance so the expected phase profile is linear at the sides. When the voltage is high enough a logarithmic lens is also expected. To check this, the applied voltages are *V*_*2*_ = 0.5 V_RMS_ and *V*_*1*_ varying from 1.5 to 2.6 V_RMS_ (see Table 2). For this simulation, all the structural parameters are considered (diameter of the inner circle and effect of the concentric electrodes on *R*_*1*_). The phase distribution is measured by using the setup commented in the previous section.

**Table 2:** Used voltages *V*_*1*_ and *V*_*2*_ and estimated *V*_*C*_ from Eq. 1.

V_1_	V_2_	V_C_	Type
1.5	0.5	0.75	Negative
1.7	0.5	0.80	
2	0.5	0.88	
2.3	0.5	0.95	
2.6	0.5	1.03	

**Figure 9:**
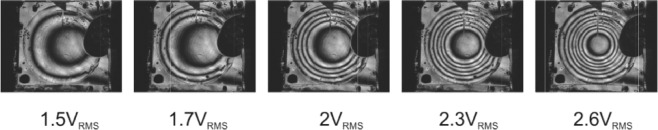
Experimental optical phase shift produced by the proposed device for several voltages at *V*_*1*_.

**Figure 10:**
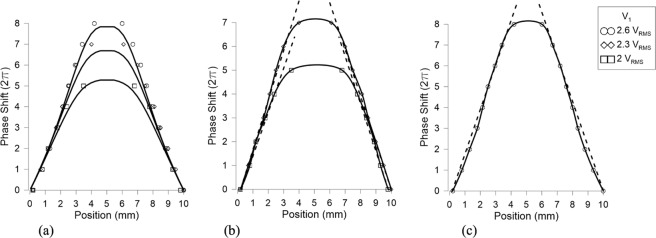
Optical phase shift produced by the proposed device for several voltages at *V*_*1*_, (**a**) experimental (dots) and simulated (lines) results, (**b**) experimental results and a straight line showing axicon like profile at the sides, (**c**) experimental results showing a logarithmic axicon like profile.

As can be seen in Fig. 9, the gap between concentric electrodes does not affect negatively the phase distribution and the optical phase shift is homogeneously distributed across all the active area. The phase variation between adjacent electrodes is negligible; this effect is not noticeable in the images of Fig. 9. In order to compare these results with the theoretical model, the phase profiles are extracted by using the technique commented in the previous section (see Fig. 10). In order to avoid the bubble defect, the profile is extracted from a diagonal line. For low voltages, the bubble effect distorts significantly the resulting phase profile; for this reason, these profiles are neither extracted nor simulated. For the simulation, a 50 µm thickness device and a LC similar to the used in the experimental device (6CHBT *Δn* = 0.17) are considered.

The graph of Fig. 10(a), shows axicon type profiles for both simulated (lines) and experimental (dots) results. Figure 10(b) show the experimental results when the voltage *V*_*1*_ is lower than 2.6 V_RMS_. In this case, the sides of the phase profile follow the straight lines. However, for a voltage of 2.6 V_RMS_ the profile is more approximated to a logarithmic axicon. As can be observed in Fig. 10(c), the phase profile does not follow the straight line. This effect is more pronounced as voltage increases. In conclusion, the transmission line is capable of producing a voltage distribution accordingly to the simulations, which is homogeneously distributed across the active area by means of concentric electrodes.

The switching time of LC devices is directly related to the rising and decay time of the LC molecules. These relations are quadratically proportional to the device thickness. In this case, the required thickness only will be dependent on the required optical power. Thickness above 50 µm usually have switching times in the order of seconds. For this reason, this device is not intended for fast switching lenses. Despite this, the proposed transmission line technique could be used to fabricate Fresnel lenses, on which no multielectrode would be required. This would solve the switching time issue, allowing high optical powers with thickness in the order of few microns. This idea has to be further investigated.

### Summary

In this work, a novel technique to create adaptive liquid crystal lenses and other optical components is proposed and demonstrated. This proposal avoids all the disadvantages of previous techniques, involving a simple fabrication process and low voltage control. Moreover, thin lenses can also be obtained. The novelty of the proposal resides in a micro-structured ITO transmission line, combined with concentric electrodes designed to distribute the voltage homogenously across the entire surface of the device. The voltage profile can be controlled from the center to the lens side, allowing a positive-negative phase shift tunability. The theoretical study reveals that an optical power of 5.7D would be possible for a device with 100 µm thick and an aperture of 1 cm. The theoretical study is also validated by experimental results. One device is designed and experimentally demonstrated. This novel structure opens new venues of research in phase-only LC optical devices.
