# Author Correction: Positive-negative tunable liquid crystal lenses based on a microstructured transmission line

**DOI:** 10.1038/s41598-020-79341-8

**Published:** 2020-12-14

**Authors:** J. F. Algorri, P. Morawiak, N. Bennis, D. C. Zografopoulos, V. Urruchi, L. Rodríguez-Cobo, L. R. Jaroszewicz, J. M. Sánchez-Pena, J. M. López-Higuera

**Affiliations:** 1 Photonics Engineering Group, University of Cantabria, 39005 Santander, Spain; 2 New Technologies and Chemistry Faculty, Military University of Technology, 00-908 Warsaw, Poland; 3 Consiglio Nazionale Delle Ricerche, Istituto Per La Microelettronica E Microsistemi (CNR-IMM), 00133 Rome, Italy; 4 Department of Electronic Technology, Carlos III University, 28911 Madrid, Spain; 5 CIBER-Bbn, Instituto de Salud Carlos III, 28029 Madrid, Spain; 6 Instituto de Investigación Sanitaria Valdecilla (IDIVAL), 39011 Santander, Spain

Correction to: *Scientific Reports* 10.1038/s41598-020-67141-z, published online 23 June 2020

This Article contains a typographical error in the Acknowledgements section.

“the Ministerio de Economía y Competitividad of Spain (TEC2013-47342-C2-2-R)”

should read:

"the Ministerio de Economía y Competitividad of Spain (TEC2016-77242-C3-1-R)"

